# Genomics-Assisted Identification and Characterization of the Genetic Variants Underlying Differential Nitrogen Use Efficiencies in Allotetraploid Rapeseed Genotypes

**DOI:** 10.1534/g3.118.200481

**Published:** 2018-07-09

**Authors:** Ying-peng Hua, Ting Zhou, Qiong Liao, Hai-xing Song, Chun-yun Guan, Zhen-hua Zhang

**Affiliations:** * Southern Regional Collaborative Innovation Center for Grain and Oil Crops in China, College of Resources and Environmental Sciences, Hunan Agricultural University, Changsha, China; † National Center of Oilseed Crops Improvement, Hunan Branch, Changsha, China

**Keywords:** Genetic variants, genotypes, nitrogen use efficiency, rapeseed, whole-genome re-sequencing

## Abstract

Nitrogen (N) is a non-mineral macronutrient essential for plant growth and development. Oilseed rape (A_n_A_n_C_n_C_n_, 2*n* = 4*x* = 38) has a high requirement for N nutrients whereas showing the lowest N use efficiency (NUE) among crops. The mechanisms underlying NUE regulation in *Brassica napus* remain unclear because of genome complexity. In this study, we performed high-depth and -coverage whole-genome re-sequencing (WGS) of an N-efficient (higher NUE) genotype “XY15” and an N-inefficient (lower NUE) genotype “814” of rapeseed. More than 687 million 150-bp paired-end reads were generated, which provided about 93% coverage and 50**×** depth of the rapeseed genome. Applying stringent parameters, we identified a total of 1,449,157 single-nucleotide polymorphisms (SNPs), 335,228 InDels, 175,602 structure variations (SVs) and 86,280 copy number variations (CNVs) between the N-efficient and -inefficient genotypes. The largest proportion of various DNA polymorphisms occurred in the inter-genic regions. Unlike CNVs, the SNP/InDel and SV polymorphisms showed variation bias of the A_n_ and C_n_ subgenomes, respectively. Gene ontology analysis showed the genetic variants were mapped onto the genes involving N compound transport and ATPase complex metabolism, but not including N assimilation-related genes. On basis of identification of N-starvation responsive genes through high-throughput expression profiling, we also mapped these variants onto some key NUE-regulating genes, and validated their significantly differential expression between the N-efficient and -inefficient genotypes through qRT-PCR assays. Our data provide genome-wide high resolution DNA variants underlying NUE divergence in allotetraploid rapeseed genotypes, which would expedite the effective identification and functional validation of key NUE-regulating genes through genomics-assisted improvement of crop nutrient efficiency.

Oilseed rape (*Brassica napus* L.), a high-value crop species, is widely cultivated and harvested for the production of vegetable oil, livestock protein meal and biodiesel (Blackshaw *et al.* 2011). The allotetraploid *B. napus* (A_n_A_n_C_n_C_n_, ∼1,345 Mb, 2*n* = 4*x* = 38) originated from spontaneous interspecific hybridization of the diploid progenitors *Brassica rapa* (A_r_A_r_, ∼485 Mb, 2*n* = 2*x* = 20) (Wang *et al.* 2011) and *Brassica oleracea* (C_o_C_o_, ∼630 Mb, 2*n* = 2*x* = 18) (Liu *et al.* 2014) ∼7,500 years ago (Chalhoub *et al.* 2014; Bayer *et al.* 2017; Sun *et al.* 2017). Approximate 70% of angiosperms, including *B. napus*, have undergone one or more chromosome doubling events during their evolutionary processes (Masterson 1994). The allopolyploidy events occurring in *B. napus* generated many duplicated segments and homeologous regions within the genome (Chalhoub *et al.* 2014), which poses great difficulty in mapping and cloning of gene(s) responsible for target agronomic traits.

Recently, with the availability of rapeseed genome sequences combined with the roaring development of high-throughput next generation sequencing (NGS) technologies, it has now become convenient to explore genetic variability at the genome-wide scale by re-sequencing diverse rapeseed genotypes. NGS has provoked a revolution in plant genomics research and offers a wide range of applications (Edwards *et al.* 2012). For example, whole-genome re-sequencing (WGS) studies have also been widely utilized in gene identification, quantitative trait locus (QTL) mapping, genome diversification, evolutionary and phylogenetic analysis (Hua *et al.* 2016a, b; Stein *et al.* 2017). Using a large set of high-density genetic polymorphisms identified by WGS, Wang *et al.* (2017) identified numerous genomic loci associated with three seed-quality traits. Apart from bridging the gap of genotype to phenotype, WGS has immense potential to unravel the functional complexity of rapeseed genome and can promote molecular breeding to improve the agronomic traits of interest.

Nitrogen (N) is a non-mineral macronutrient essential for plant growth and development (Konishi and Yanagisawa 2014). In most plants, inorganic N is acquired by roots in the form of NO_3_^-^. For many species, NO_3_^-^ is not assimilated in the roots, but is secreted into the xylem sap for long-distance translocation to the shoot, where it enters the cells to be metabolized and/or stored in the vacuoles. Therefore, to reveal the genetic basis underlying differential N use efficiencies (NUEs), we focused on the genes responsible for efficient NO_3_^-^ uptake and translocation.

Several plasma membrane transporters involved in NO_3_^-^ influx into the cell have been identified in *Arabidopsis thaliana* (Wang *et al.* 2012), especially in the roots where the NPF (NRT1/PTR Family, Léran *et al.* 2014) members and NRT2 transporters are predominantly implicated. Among them, NRT1.1 and NRT2.1 (together with its partner protein NAR2.1) are the major dual- and high-affinity contributors to efficient NO_3_^-^ influx into root cells. AtNRT2.4 is also involved in root NO_3_^-^ uptake at very low NO_3_^-^ concentrations and in shoot phloem NO_3_^-^ loading (Kiba *et al.* 2012). AtNRT2.5 is essential for efficient NO_3_^-^ uptake and remobilization in adult plants by participating in phloem NO_3_^-^ loading under severe N starvation (Lezhneva *et al.* 2014). When NO_3_^-^ enters root cells, NPF7.3/NRT1.5 mediates efflux of NO_3_^-^ to the xylem vessels, and NPF7.2/NRT1.8 retrieves NO_3_^-^ from the xylem sap into xylem parenchyma cells (Lin *et al.* 2008; Li *et al.* 2010; Chen *et al.* 2012). Currently, Liu *et al.* (2017) found that Nitrogen Limitation Adaptation (NLA) is involved in source-to-sink remobilization of NO_3_^-^ by mediating the degradation of NRT1.7 in *Arabidopsis*. Taken together, NUE is a complex trait controlled by multiple genes implicated in NO_3_^-^ uptake, translocation and remobilization. The recent OsNRT1.1A and OsNRT1.1B identified in rice highlight the crucial role of efficient NO_3_^-^ uptake in NUE regulation (Hu *et al.* 2015; Wang *et al.* 2018b).

*B. napus* has a higher nutrient requirement for optimum seed yield than cereals (Grant and Bailey 1993), and it is extremely susceptible to N deficiency (Rathke *et al.* 2005). Conventional crop management practices require the use of relatively high amounts of N fertilizers (from 150 to 300 kg of N hm^-2^) to ensure an optimum yield (Rathke *et al.* 2006). Significant differences in NUEs, including N uptake efficiencies, N remobilization efficiencies and N assimilation efficiencies, among others, have been observed in allotetraploid rapeseed genotypes (Schulte auf’m Erley *et al.* 2007; Wang *et al.* 2014; Sorin *et al.* 2016). However, the molecular mechanisms underlying efficient N uptake, transport and utilization remain poorly understood because of the rapeseed genome complexity. However, for the model crop rice (genome size: ∼450 Mb), WGS has contributed to the genetic basis identification of NUE variations between the two main subspecies (*indica* and *japonica*) of Asian cultivated rice (Hu *et al.* 2015). Genetic diversity for NUEs in rapeseed genotypes can be therefore, investigated using high-throughput NGS-based WGS of genotypes with contrasting NUEs.

In the present study, we carried out WGS of a low-N tolerant (N-efficient, higher NUE) genotype “Xiang-you 15” and a low-N sensitive (N-inefficient, lower NUE) genotype “814” of rapeseed on an Illumina HiSeq 4000 NGS platform. Further, we have identified numerous genomic variants, including 1,449,157 single nucleotide polymorphisms (SNPs), 335,228 insertions/deletions (InDels), 175,602 structure variations (SVs) and 86,280 copy number variations (CNVs), in these genotypes at a genome-wide scale. Combining the WGS and transcriptome sequencing data, we also evaluated functional significance of these polymorphic sites by correlating their presence (structural and functional annotations) in the NUE-regulating genes responsive to N limitation. What is noteworthy, the genome-wide high resolution SNP and InDel sites, discovered from the rapeseed genotypes with differential NUEs, would accelerate the identification and functional validation of key NUE-regulating genes for genomics-assisted crop improvement.

## Materials and Methods

### Plant materials and hydroponic culture

In previous studies, we have identified an N-efficient genotype “Xiang-you 15” (“XY15”) and an N-inefficient genotype “814” of rapeseed (Han *et al.* 2015a, b; Han *et al.* 2016). For isolation of genomic DNA (gDNA) and total RNA, the *B. napus* seedlings were hydroponically cultivated according to the method described by Hua *et al.* (2016a, b). Plump seeds of a similar size were surface sterilized and then germinated on a piece of moist gauze immobilized on a black plastic tray. Uniform *B. napus* seedlings with similar hypocotyl and root lengths and cotyledon sizes after germination were transplanted into black plastic containers holding 10 L Hoagland solution. The nutrient solution was constantly aerated throughout the experiments and refreshed every 3 d, with one-quarter-strength solution initially added and increasing to one-half strength and eventually full strength.

For RNA sequencing used for the identification of genes responsive to N limitation, the rapeseed seedlings were cultivated under high NO_3_^-^ (6.0 mM) for 10 d, and then transferred to low NO_3_^-^ (0.30 mM). At 0 h, 3 h and 72 h, the shoots and roots of the seedlings were individually sampled. A total of 27 RNA samples were subjected to an Illumina Hiseq X Ten platform (Illumina Inc., San Diego, CA, USA), which generated approximate 6.0-Gb of sequencing data with 150-bp paired-end (PE) reads for each sample. Transcript abundances (FPKM values) were determined from the RNA-seq data with the method described by Hua *et al.* (2017). Three independent biological replicates for each treatment were prepared for the high-throughput transcriptomic profiling.

For quantitative reverse-transcription PCR (qRT-PCR) assays, the solution concentration of N was adjusted to 1.0 mM and 5.0 mM by reducing KNO_3_ and replacing Ca(NO_3_)_2_ by CaCl_2_, while K^+^ was complemented by adding K_2_SO_4_ (Wang *et al.* 2017). The seedlings were cultivated in an illuminated growth chamber (300-320 μmol m^-2^ s^-1^; 24° daytime: 22° night; 16 h photoperiod) for 15 d.

### DNA isolation and genome sequencing

High quality gDNA was extracted from fresh young leaves of the N-efficient genotype “XY15” and the N-inefficient genotype “814” with the cetyltrimethylammonium bromide (CTAB) mini-prep method (Murray and Thompson 1980). Integrity of gDNA was assessed by Bioanalyzer 2100 (Agilent Technologies, Singapore). WGS of the two rapeseed genotypes was performed on an Illumina HiSeq4000 (paired-end, read length = 150 bp) (Illumina Inc., San Diego, CA, USA) by Novogene Technologies (Beijing, China). Sample preparation and sequencing were performed according to the standard Illumina protocol (https://www.illumina.com/?langsel=/us/) as follows: quantified gDNA was treated with the ultrasonic wave to produce DNA fragments, which was purified using the QIA quick PCR kit. End repair was performed with poly-A on the 3′ends, then the adaptors were ligated, agarose gel electrophoresis was used to select fragments for PCR amplification. Sequencing was performed through establishing a library with Illumina HiSeq4000. The reads were aligned using the Burrows-Wheeler transformation. Low-quality reads with adaptor sequences and duplicated reads were filtered, and the remaining high-quality data were used in the sequence mapping.

### Mapping of high quality clean reads to the reference genome

Read quality check and alignment were performed according to the standard Illumina analysis pipeline (https://www.illumina.com/?langsel=/us/). The adapter and low quality sequence reads were discarded and high quality sequences with mapping quality (MAPQ) **≥** 20 were retained for read alignment. Prior to alignment, raw reads were first trimmed based on the quality, compositions, and adapter sequences of nucleotides. After modulation, reads **<** 30 bp was removed for further analysis. The trimmed reads were aligned to the rapeseed genome reference (http://brassicadb.org/brad/datasets/pub/Genomes/Brassica_napus/).

### Genome-wide calling of SNP, InDel, SV and CNV variants

The SAM tools software (v 0.1.18) was utilized to detect SNPs and InDels and investigate their quantity, type and distribution on chromosomes and gene coding regions. SVs and CNVs were investigated using the Control-FREEC software (FREEC v6.2) (Boeva *et al.* 2012).

### Functional characterization of genetic variants

A gene ontology (GO) analysis of the genetic variants was performed by the PANTHER Classification System (http://www.pantherdb.org/data/) (Mi *et al.* 2005). The enriched GO items were illustrated with the word cloud generator WordArt (https://wordart.com/). Using the gene sequences from *A. thaliana* as seed sequences, a BLAST analysis was conducted to search for homolog sequences of rapeseed in the *Brassica* Database v. 1.1 (http://brassicadb.org/brad/) (Wang *et al.* 2015).

Gene co-expression networks were constructed to identify gene interactions and locate core genes that connect most neighboring genes involved in N starvation response in *B. napus*. The threshold of Pearson correlation values was set as the default parameters (http://plantgrn.noble.org/DeGNServer/Analysis.jsp), and then the gene co-expression networks were constructed with CYTOSCAPE v. 3.2.1 (http://www.cytoscape.org/) (Kohl *et al.* 2011).

### Quantitative reverse-transcription PCR (qRT-PCR) assays

The qRT-PCR assays were used to identify the differential gene expression between the N-efficient and N-inefficient rapeseed genotypes. After the treatment of RNase-free DNase I with RNA samples, total RNA was used as the templates for complementary DNA (cDNA) synthesis with the PrimeScript RT reagent Kit with gDNA Eraser (Perfect Real Time) (TaKaRa, Shiga, Japan). The qRT-PCR assays for the detection of relative gene expression were performed using SYBR *Premix Ex Taq* II (Tli RNaseH Plus) (TaKaRa, Shiga, Japan) under an Applied Biosystems StepOne Plus Real-time PCR System (Thermo Fisher Scientific, Waltham, MA, USA). The thermal cycles were as follows: 95° for 3 min, followed by 40 cycles of 95° for 10 s, then 60° for 30 s. Melt curve analysis to ensure the primer gene-specificity was conducted as follows: 95° for 15 s, 60° for 1 min, 60°-95° for 15 s (+0.3° per cycle). Expression data were normalized using the public reference genes *BnaEF1-α* (Maillard *et al.* 2016) and *BnaGDI1* (Yang *et al.* 2014), and relative gene expression was calculated with the 2^-ΔΔC^*_T_* method (Livak and Schmittgen 2001).

### Data availability

The raw sequences of whole-genome re-sequencing and transcriptome sequencing are available at the short-read archive (SRA) of the National Biotechonology Centre of Information (NCBI) under the BioProject ID PRJNA340053. Supplemental material available at Figshare: https://doi.org/10.25387/g3.6724913.

## Results

### Overview of the WGS data of the N-efficient and N-inefficient genotypes

**Figure 1:**
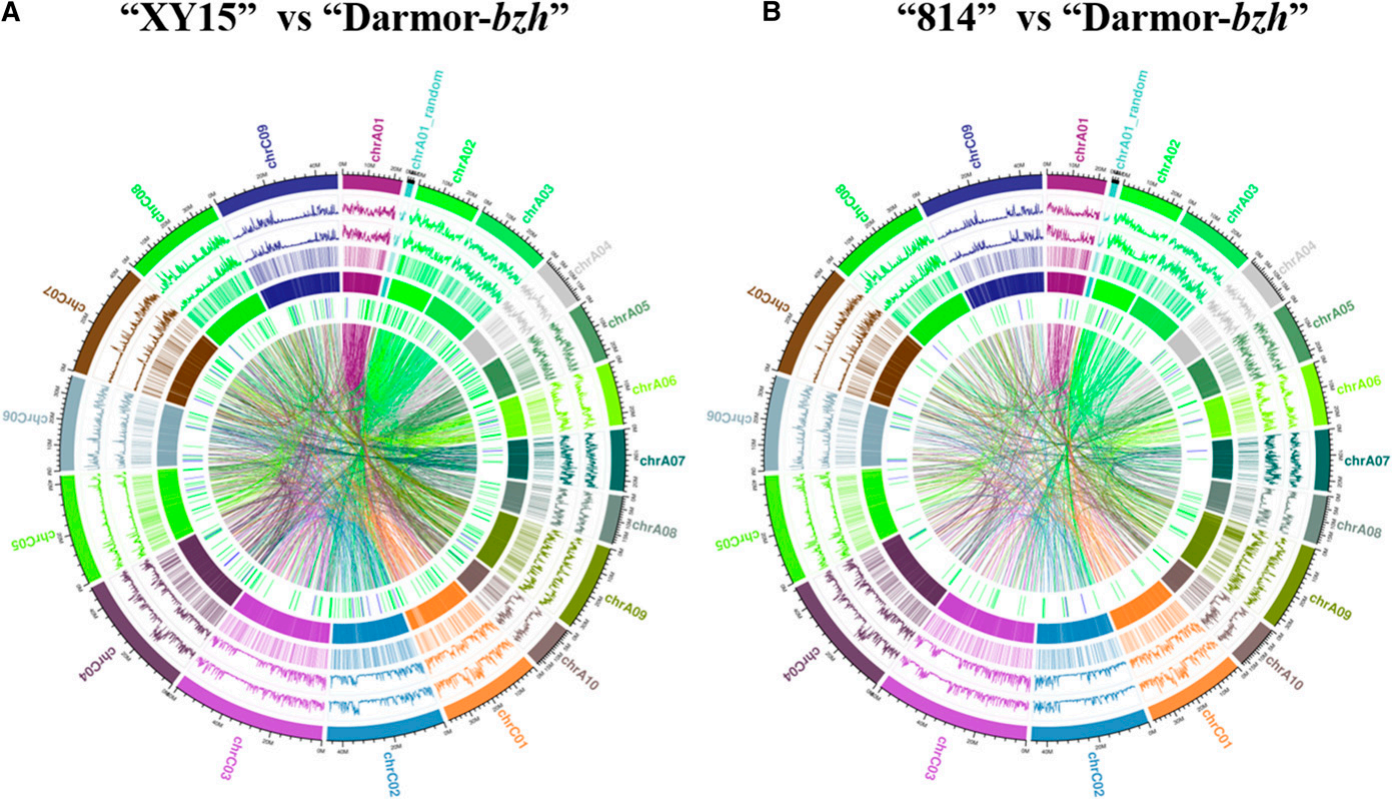
Overview of the genetic diversity between the whole-genome sequenced rapeseed cultivars (N-efficient cv. “XY15” and N-inefficient cv. “814”) and the *de novo* sequenced reference genome of “Darmor-*bzh*”. Overview of the genome-wide genetic variants between “XY15” *vs.* “Darmor-*bzh*” (A) and “814” *vs.* “Darmor-*bzh*” (B), which are delineated by the Circos program. In the Circos figure, the variants are as follows outside-to-inside: chromosomes (i), single nucleotide polymorphisms (SNPs) (ii), insertions/deletions (InDels) (iii), copy number variation (CNV) duplications (iv), CNV deletions (v), structure variations (SV; red: insertions; green: deletions; blue: inversion) (vi), and intra-/inter-chromosomal translocation (vii).

Whole genome re-sequencing of the N-efficient genotype “XY15” and the N-inefficient genotype “814” revealed the occurrence of gDNA polymorphisms at a genome-wide scale and their probable effect on differential low-N tolerance in these genotypes. In this study, we obtained ∼379 million reads in “XY15” and ∼308 million reads in “814”, respectively, with ∼98.6% high quality sequences (Q_30_ passed quality score) (Table 1). The obtained average read depth and coverage were 59.1 **×** and 94.2% for “XY15” and 49.2 **×** and 93.2% for “814”, respectively, which can also be seen in the Circos figure (Figure 1). Such a high coverage and read depth indicates the high quality assembly and sequencing data. To avoid contamination with chloroplastic and mitochondrial DNA reads, all the reads showing a depth higher than 128**×** were removed from subsequent analysis (Subbaiyan *et al.* 2012). As shown in Figure 1, thousands of genomic variants between the N-efficient “XY15” and the N-inefficient “814”, including 1,449,157 SNPs, 335,228 InDels, 175,602 CNVs and 86,280 SVs, were randomly distributed across the 19 chromosomes of *B. napus*.

**Table 1:** Overview of the whole-genome re-sequencing data of the N-efficient genotype “XY15”and the N-inefficient genotype “814”

Sample	Raw base (bp)	Clean base (bp)	Effective rate (%)	Error rate (%)	Q_20_ (%)	Q_30_ (%)	GC (%)	Mapped reads	Total reads	Mapping rate (%)	Average depth (×)	Coverage (%)
XY15	57,028,386,900	56,880,831,300	99.74	0.04	96.68	94.78	38.62	373,951,565	379,205,542	98.61	59.14	94.22
814	46,338,658,800	46,208,492,700	99.72	0.04	96.78	94.87	38.73	303,855,965	308,056,618	98.64	49.25	93.42

### Genome-wide identification and characterization of SNPs

**Figure 2:**
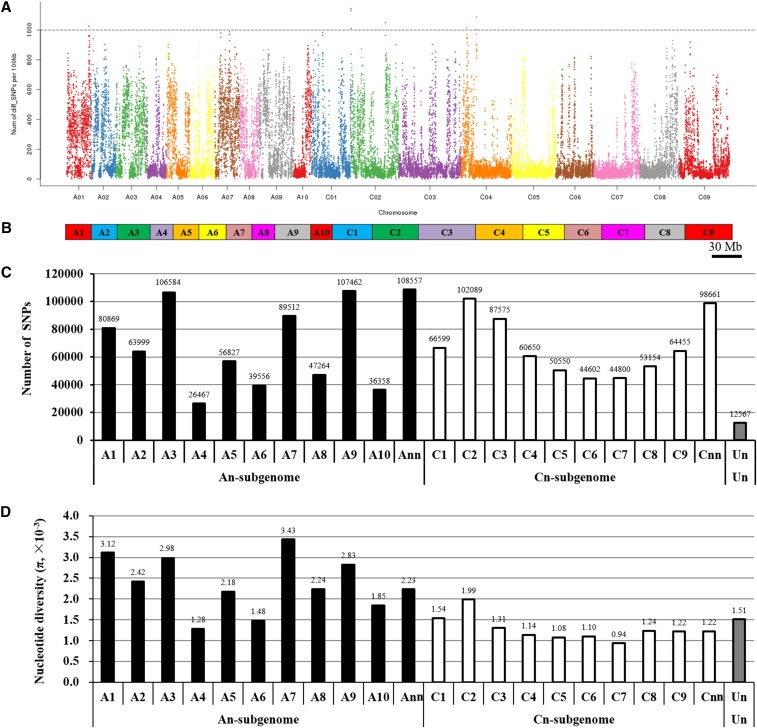
Genomic distribution and frequency of single nucleotide polymorphisms (SNPs) between the N-efficient genotype “XY15” and the N-inefficient genotype “814”. (A) Genomic distribution of SNPs. The x-axis represents the *B. napus* chromosome sizes (Mb), while the y-axis represents the number of SNPs present at that point on each chromosome. (B) Graph delineating chromosome sizes of *B. napus*. (C) Number of SNPs on each chromosome. (D) Nucleotide diversity (π) on each chromosome. “Ann” or “Cnn” represents the genome scaffolds anchored to the A_n_ or C_n_ subgenome but not anchored to specific chromosomes; ‘Un’ represents the genome scaffolds whose locations are unknown.

SNPs are the most common type of genetic variation. A total of 1,449,157 SNPs were identified across the 19 chromosomes of *B. napus*, with 763,455 and 673,135 SNPs distributed on the A_n_ and C_n_ subgenomes, respectively (Figure 2A, B), showing biased subgenome polymorphisms. The SNPs appeared non-randomly distributed among different chromosomes, but also within each chromosome. In general, most of the SNPs were frequent in the distal parts of chromosomes (Figure 2A, B), which may correspond to genomic regions with higher recombination frequency and gene density. The SNP numbers were not correlated with the length of chromosomes or with gene density. The number of SNPs ranged from 26,467 (chr.A4) to 107,462 (chr.A9) with an average of 76,271 SNPs on each chromosome (Figure 2C). The nucleotide diversity π (average number of SNPs per nucleotide) varied from 1.04 **×** 10^−3^ (chr.C5) to 3.97 **×** 10^−3^ (chr.A5), with an average value of π **=** 2.37 **×** 10^−3^ on the A_n_ subgenome and π = 1.28 **×** 10^−3^ on the C_n_ subgenome, respectively. There were a total of eight SNP hot-spot regions identified on the A1 (one), C2 (four) and C4 (three) chromosomes, where **>** 1,000 SNPs occurred within a 1-kb genomic region (Jain *et al.* 2014). In contrast, a sum of 623 and 2378 SNP cold-spot regions, where **<** 20 SNPs occurred within a 1-kb genomic region (Jain *et al.* 2014), were detected on the A_n_ and C_n_ subgenomes, respectively.

**Figure 3:**
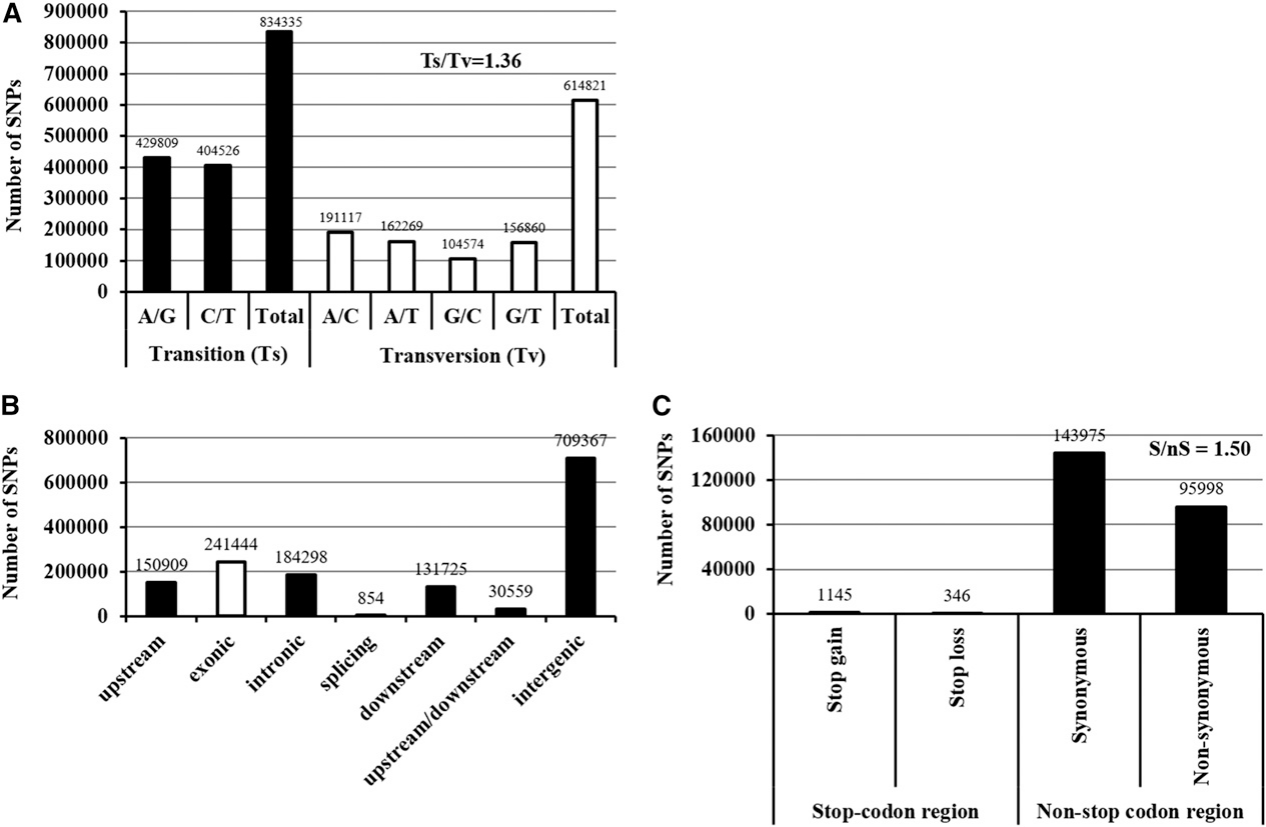
Annotation of single nucleotide polymorphisms (SNPs) identified between the N-efficient genotype “XY15” and the N-inefficient genotype “814”. (A) Transition (Ts) and transversion (Tv) frequency of the SNPs. (B) Genic distribution of the SNPs. (C) Number of the synonymous (S) and non-synonymous (nS) SNPs. The upstream and downstream regions are defined as the 2.0-kb upstream intervals of the start codon (ATG) and the 1.0-kb downstream of the stop codons. The upstream/ downstream regions refer to the overlapped genomic intervals of the upstream and downstream areas between two neighboring genes.

The detected SNPs were categorized into two groups: transitions (A/G and C/T; Ts) and *trans*-versions (A/C, A/T, C/G and G/T; Tv) based on the nucleotide variations between the N-efficient genotype “XY15” and the N-inefficient genotype “814”. Among the 1,449,157 SNPs, 834,335 (57.57%) SNPs belonged to the transition type, which was more than that of the *trans*-versions (614,821, 42.43%) (Figure 3A). Concerning the transitions, the frequency of A/G type was higher than that of C/T; however, among the *trans*-versions, the frequency of the A/C sub-type (191,117, 31.08%) was in the largest numbers and G/C was the lowest (17.01%) (Figure 3A). The ratio of Ts to Tv were approximately 1.36, which was larger than the expected value (0.5).

We then used the annotations of the reference “Darmor-*bzh*” genome to examine the distribution of SNPs within various genomic features. There were a sum of 426,596 SNPs, accounting for 29.44% of the total SNPs, detected in the genic regions (including exonic, intronic and splicing areas), whereas a larger proportion of SNPs (70.56%) were identified in the 2.0-kb upstream (promoter), 1.0-kb downstream, and other inter-genic regions. Within the SNPs located in the genic areas, more than half (241,444; 56.60%) the variations occurred in the coding sequences (Figure 3B). Non-synonymous SNPs that lead to amino acid changes in the protein products are more likely to lead to functional alterations, which may further affect plant performance. Therefore, we analyzed the effect of SNPs on amino acid substitution. In general, only a very small fraction (0.61%) of the SNPs occurring in the exons were mapped onto the stop codons (Figure 3C). Among these, 1,145 non-synonymous SNPs transformed stop codons to amino acid codons, whereas 346 non-synonymous SNPs transformed amino acid codons to stop codons. Among the SNPs mapped onto the non-stop codon regions, 143,975 SNPs did not result in alterations in the amino acid sequences, which were more than the non-synonymous types (95,977) (Figure 3C). The ratio of synonymous SNPs to non-synonymous SNPs was close to 1.50, which was similar to the result of our previous study (Hua *et al.* 2016a, b). The SNPs identified in this study causing non-synonymous amino acid substitutions can also be utilized to directly identify causal genes responsible for rapeseed NUE variations in association studies.

### Genome-wide identification and characterization of InDels

**Figure 4:**
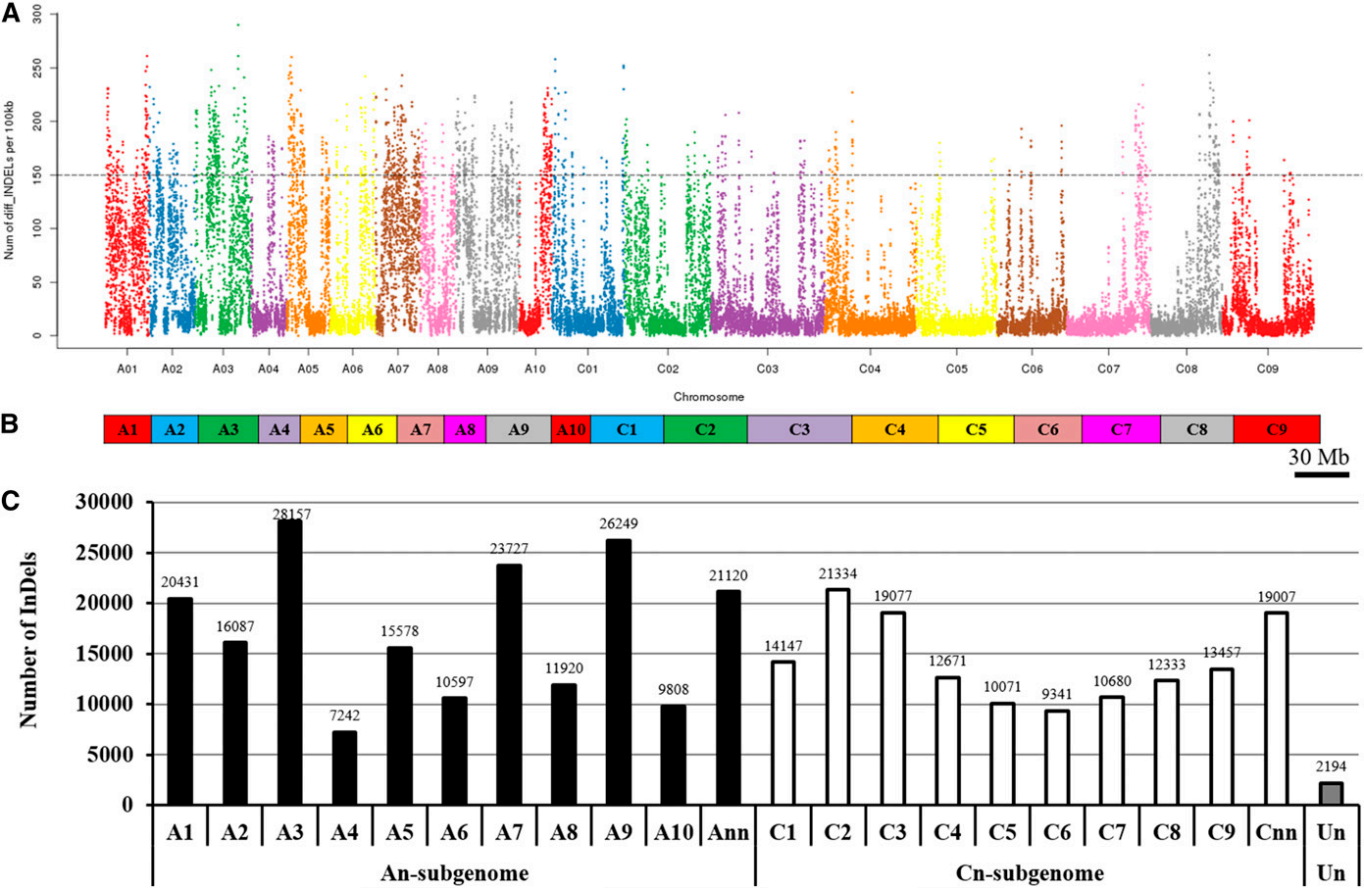
Genomic distribution and frequency of insertions/deletions (InDels) between the N-efficient genotype “XY15” and the N-inefficient genotype “814”. (A) Genomic distribution of InDels. The x-axis represents the *B. napus* chromosome sizes (Mb), while the y-axis represents the number of InDels present at that point on each chromosome. (B) Graph delineating chromosome sizes of *B. napus*. (C) Number of InDels on each chromosome. “Ann” or “Cnn” represents the genome scaffolds anchored to the A_n_ or C_n_ subgenome but not anchored to specific chromosomes; “Un” represents the genome scaffolds whose physical locations are unknown.

**Figure 5:**
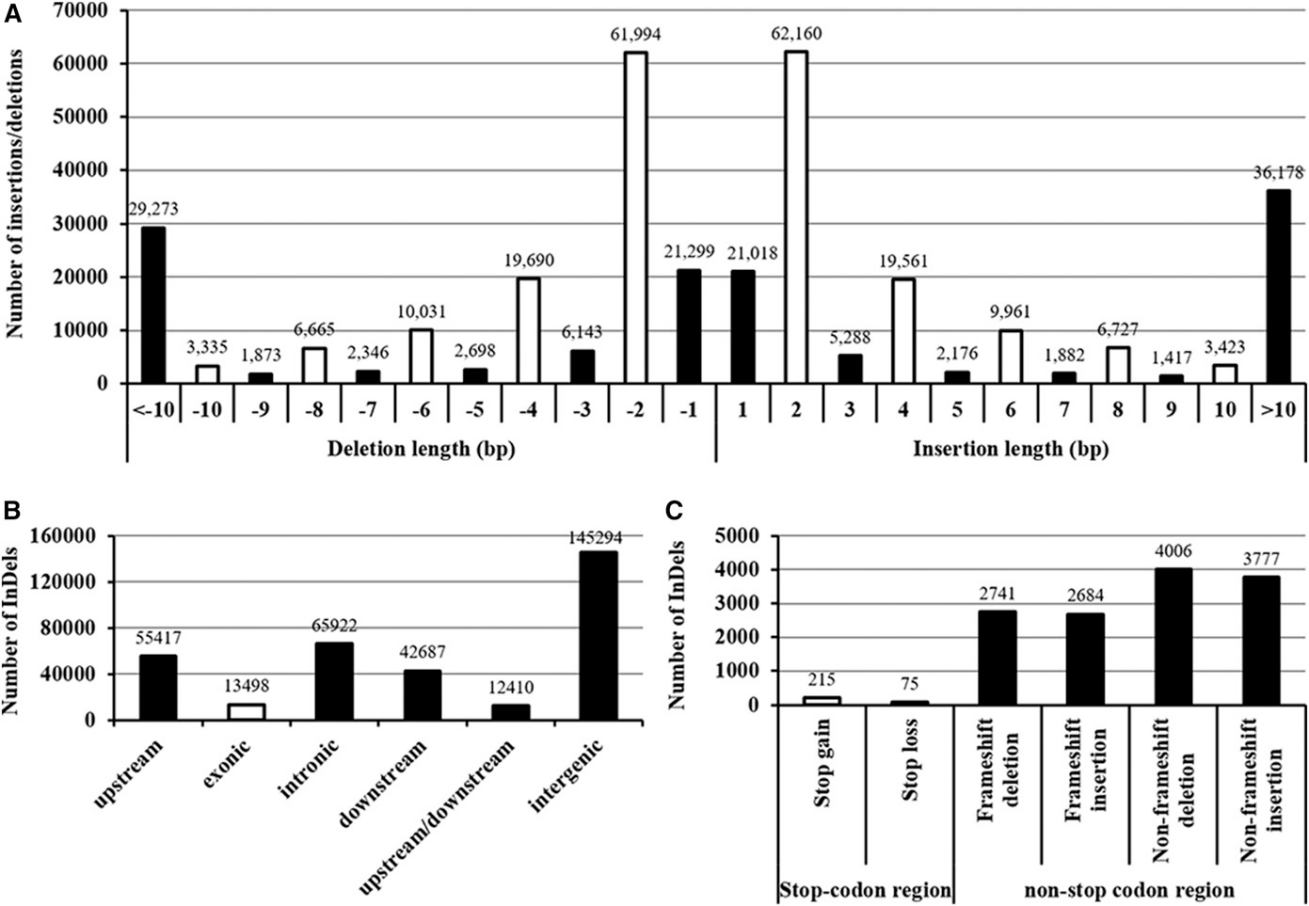
Annotation of insertions/deletions (InDels) identified between the N-efficient genotype “XY15” and the N-inefficient genotype “814”. (A) The frequency distribution of different InDel sizes. The x-axis represents the InDel sizes (bp): the positive and negative values indicate nucleotide insertion and deletion in “XY15” relative to “814”, respectively. (B) Genic distribution of the InDels. (C) The frequency distribution of large-effect InDels in the coding regions.

The WGS data revealed 335,228 InDels unevenly distributed over the *B. napus* genome (A1-A10, C1-C9) (Figure 4A-B), ranging from 7,242 (chr. A4) to 17,641 (chr. A3) (Figure 4C). Similarly to the SNPs, the InDels on the A_n_ subgenome were also more than those on the C_n_ subgenome (Figure 4C). The lengths of InDels ranged from mono-nucleotide to poly-nucleotide (> 10), whereas the frequency of which was not closely correlated with the variation length of nucleotides (Figure 5A). In terms of the insertions and deletions with same lengths, the numbers were similar (Figure 5A), which suggested the random occurrence of InDels between the N-efficient and N-inefficient genotypes. Di-nucleotide InDel (124,154) was the most frequent type, which accounted for more than one-third (37.04%) of the total InDels (Figure 5A). Different from previous studies (Hua *et al.* 2016b; Jain *et al.* 2014), we found that the InDels with even-length variations were remarkably dominant over than the odd-length variants (Figure 5A). Similar to the genomic distribution of SNPs, the majority of total InDel variants also occurred in the 2.0-kb upstream (promoter), 1.0-kb downstream, and other inter-genic regions, whereas fewer than one-fourth InDels (79,420; 23.69%) were identified in the genic sequences (Figure 5B). Among the InDels occurring in the exons, only about two percent (290) were identified in the stop-codon regions. For the other InDels, a total of 5425 (40.19%) caused frame-shift deletion or insertion (Figure 5C).

### Genome-wide identification and characterization of SVs

**Figure 6:**
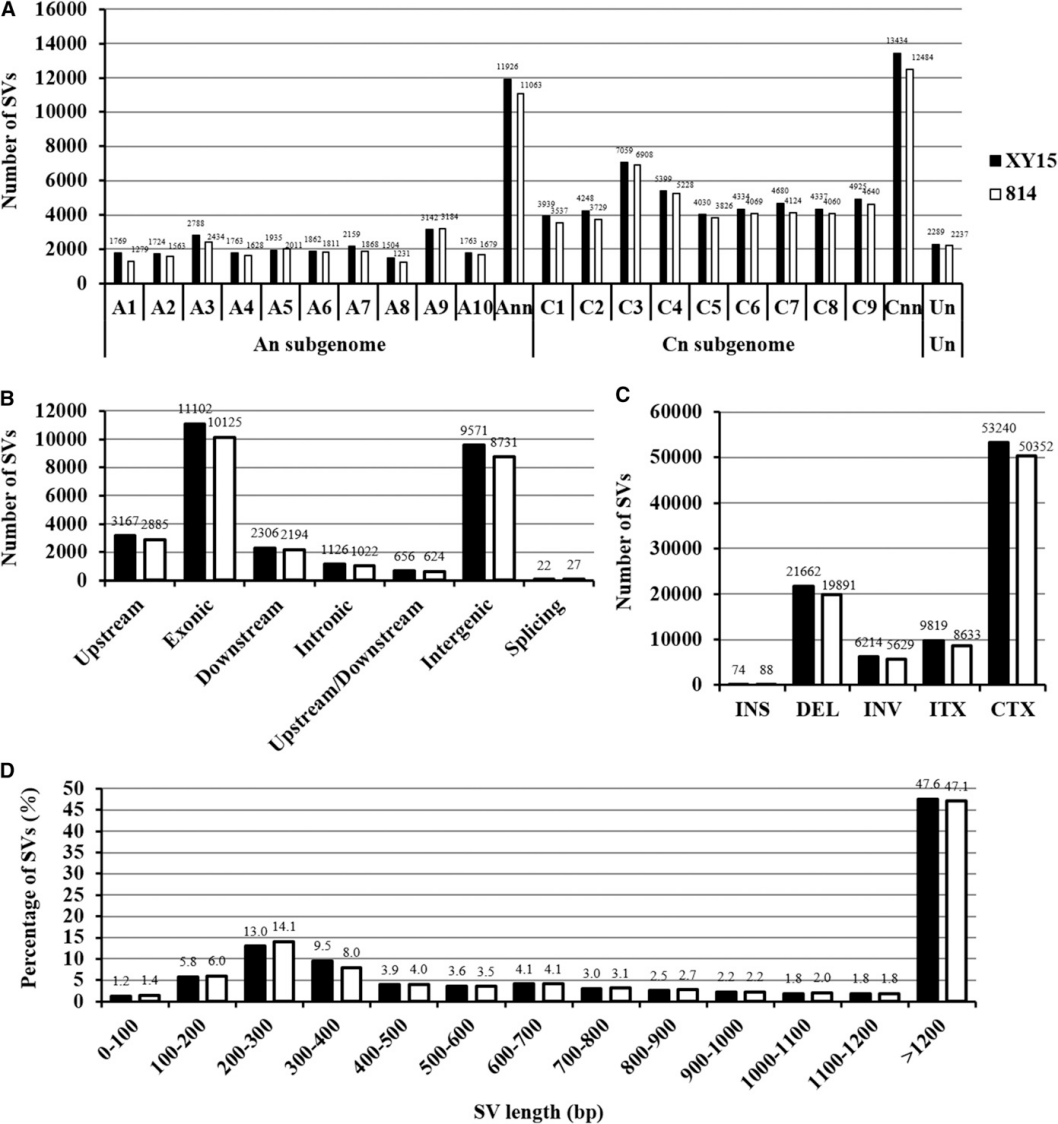
Genomic annotation of structure variations (SVs) of the N-efficient genotype “XY15” and the N-inefficient genotype “814” compared with the rapeseed genome reference “Darmor*-bzh*”. (A) Number of SVs on each chromosome. “Ann” or “Cnn” represents the genome scaffolds anchored to the A_n_ or C_n_ subgenome but not anchored to specific chromosomes; “Un” represents the genome scaffolds whose physical locations are unknown. (B) Genic distribution of SVs. (C) Number of different SV types. INS: insertion; DEL: deletion; INV: inversion; ITX: intra-chromosomal translocation; CTX: inter-chromosomal translocation. (D) Frequency distribution of SV length.

Structural variations (SV) are generally defined as genomic alterations that affect >50 bp of DNA, which are shown to have a significant effect on evolution and species phenotype. In recent years, the advent of WGS makes it feasible to study these variants in depth. Contrary to SNPs and InDels, the numbers of SVs spanning across the A_n_A_n_C_n_C_n_ genome were closely correlated with the chromosome sizes. In general, the SVs on the C_n_ subgenome were significantly more than those on the A_n_ subgenome. Specifically, the A9 of A_n_ and C3 of C_n_ chromosomes had the most two enriched SVs (Figure 6A). In terms of the genic distribution of SVs, the coding regions, which would likely to have a more influential effect on phenotype than other genic regions, possessed the most SVs (Figure 6B). The types of SVs can mainly be divided into five terms: genomic fragment insertion, deletion, inversion, intra-chromosomal translocation and inter-chromosomal translocation (Figure 6C). Obviously, most SVs led to inter-chromosomal rearrangement, followed by chromosomal fragment deletion, whereas chromosomal insertion was the least (Figure 6C). Among the SVs < 1.0 kb, those less than 100 bp was the least (1.2–1.4%) category, and the SV length (13.0–14.1%) ranging from 200-300 bp was the most (Figure 6D).

### Genome-wide identification and characterization of CNVs

Copy number variants (CNVs) are a class of SVs and are defined as fragments of DNA that are present at variable copy number in comparison with a reference genome. CNVs can create new genes, alter gene dosage and reshape gene structures. They are considered to be major sources of genetic variation, and may influence phenotypic variation, gene expression and fitness (Yu *et al.* 2011).

**Figure 7:**
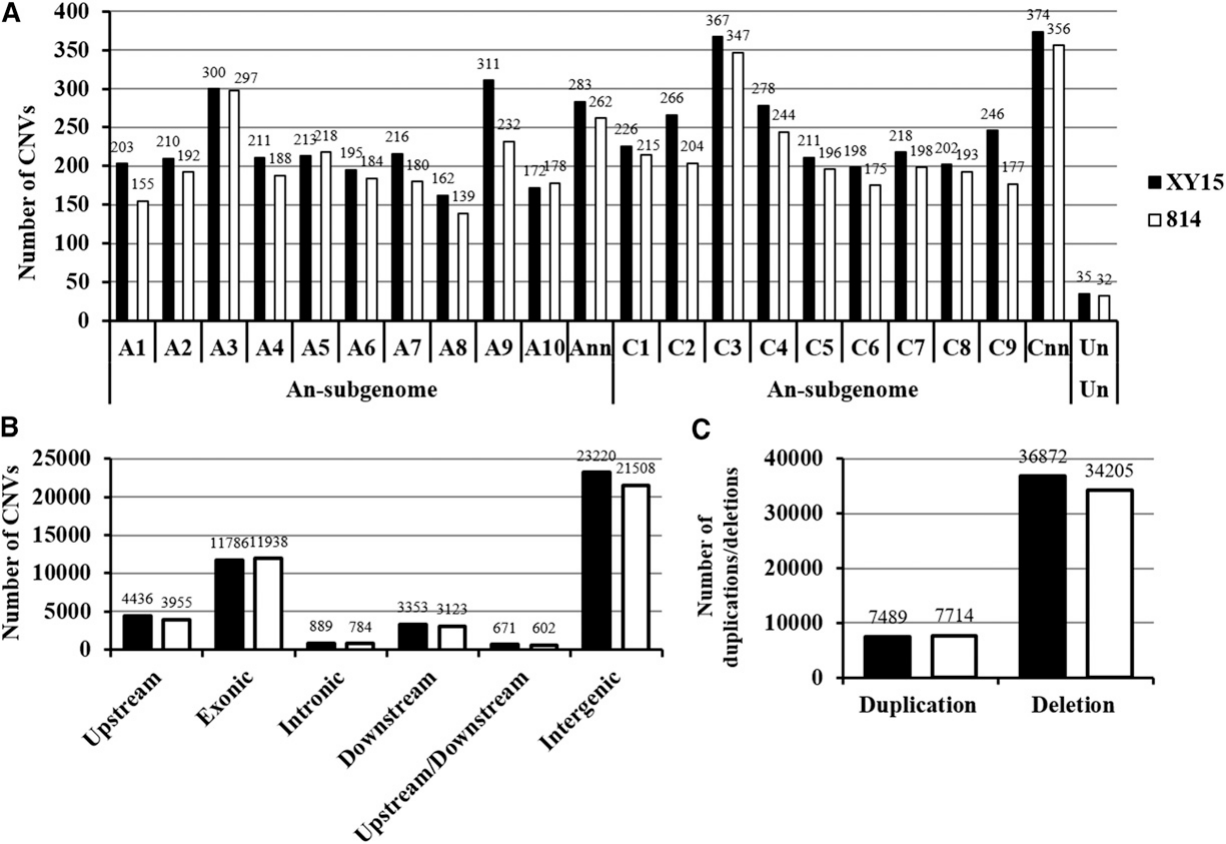
Genomic annotation of copy number variations (CNVs) of the N-efficient genotype “XY15” and the N-inefficient genotype “814” compared with the rapeseed genome reference “Darmor*-bzh*”. (A) Number of CNVs on each chromosome. “Ann” or “Cnn” represents the genome scaffolds anchored to the A_n_ or C_n_ subgenome but not anchored to specific chromosomes; “Un” represents the genome scaffolds whose physical locations are unknown. (B) Genic distribution of CNVs. (C) Number of different CNV types.

Different from the other genetic variants, the CNVs did not show biased chromosomal distribution between the A_n_ and C_n_ subgenomes (Figure 7A). The numbers of CNVs were approximately 200 on each chromosome, although the chromosome sized varied from 17.4 Mb (A10) to 60.4 Mb (C3) (Figure 7A). Similarly to SVs, the two most CNVs occurred in the exonic and intergenic (other than 1.0 kb up- and down-stream) regions (Figure 7B). The CNVs contain two types: duplication and deletion (Figure 7C). In this study, copy number deletions of genes were more dominant over gene duplications in both the N-efficient and N-inefficient rapeseed genotypes (Figure 7C).

### Gene ontology (GO) analysis of the genetic variants

The GO enrichment analysis of functional significance allowed us to distinguish major biological functions of the genes with variants between the N-efficient and N-inefficient genotypes, further contributing to our understanding of the genetic basis underlying their differential NUEs.

**Figure 8:**
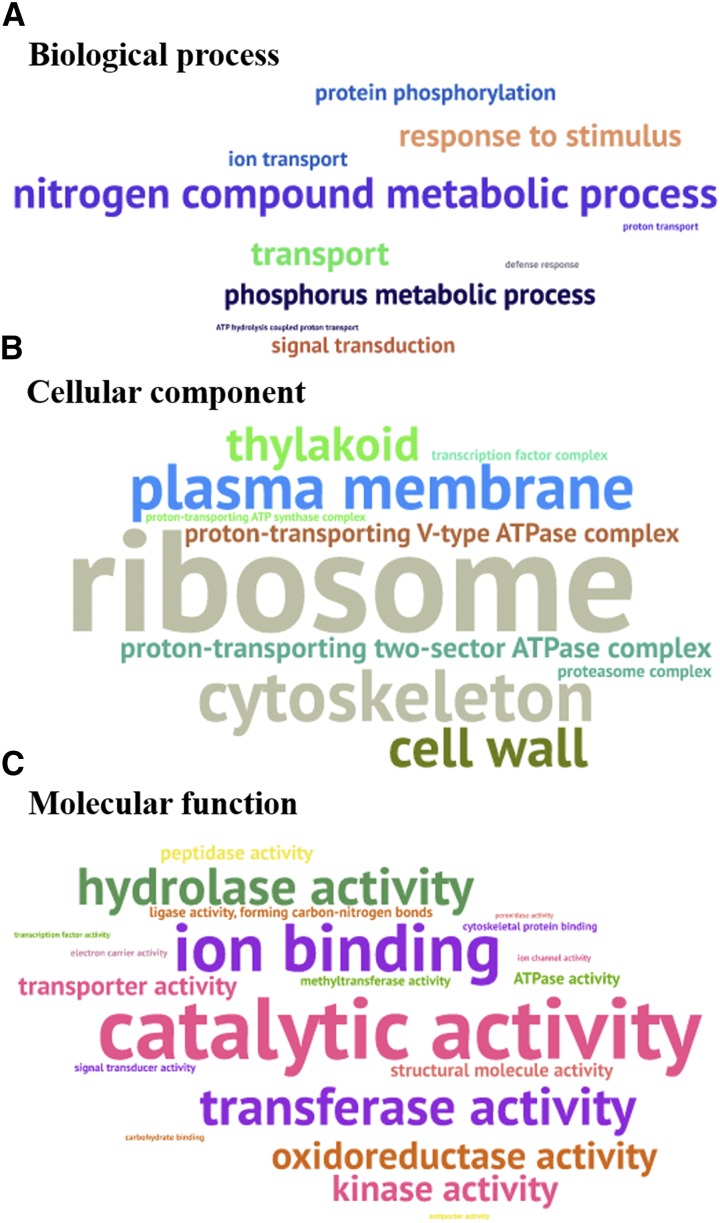
Enrichment analysis of gene ontology (GO) terms of the genes detected with polymorphisms between the N-efficient genotype “XY15” and the N-inefficient genotype “814”. Over-presentation of the biological process (A), cellular component (B) and molecular function (C) terms in the genes, which are delineated by the WordArt program. The bigger the font size, the more the corresponding GO terms.

The GO terms could be grouped into the following three categories: biological process (BP), cellular component (CC) and molecular function (MF) (Figure 8). In the BP annotations, the nitrogen compound complex process was the most enriched, which was followed by the term of response to stimulus (Figure 8A). In the GO term for CC, in addition to the usual macromolecular complex, such as cytoskeleton and cell wall, much more attention was paid to the proton-transporting V-type and two-sector ATPase complexes (Figure 8B), which provide energy and proton gradients for ion (including inorganic NO_3_^-^) active transport across the membranes (Han *et al.* 2016). In the MF category, the activities of catalase, ion binding, transferase, hydrolase, oxidoreductase, transporter, and kinase were the seven strongest GO enrichments (Figure 8C).

### Transcriptomics-assisted identification of genetic variants occurring in the genes regulating NUEs

Molecular mechanisms for NUEs are orchestrated by a set of NSR genes, such as those responsible for NO_3_^-^ uptake, translocation (mainly the NRT1 and NRT2 family members) and assimilation (mainly NO_3_^-^ reductase and glutamine synthetase genes). So, we identified the genetic variants occurring in the genes involved in N metabolism. However, we found that almost all of the genomic variations were mapped onto the NRT family genes but bot the NR and GS family genes. The results indicated that the differential NUEs between the two rapeseed genotypes used in the present study were predominantly attributed to their distinct NO_3_^-^ uptake and transport abilities, which was consistent with our previous study (Han *et al.* 2016).

To accelerate the identification of genetic variants occurring in the genes regulating NUEs, we made a high-throughput transcriptome sequencing (RNA-seq) to characterize the key genes responsive to limited N stresses. According to the normalized expression results between each two biological replicates, *Pearson* correlation coefficients (R) were calculated, most of which were more than 0.90 between each pair of biological replicates (Figure S1A). The above results indicated that the RNA-seq sequencing data were of good quality. Subsequently, we were aimed at the in-depth identification of the differential gene expression profiling of *B. napus* under short- and long-term N limitations. In the shoots, a total of 3,279 and 4,346 genes were identified to be differentially expressed at 3 h and 72 h, respectively; in the roots, more DEGs were characterized, particularly at 72 h (Figure S1B). An intersection analysis through Venn diagram indicated that there were 119 genes were simultaneously in both shoots and roots at 3 h and 72 h (Figure S1B). The gene ontology (GO) enrichment analysis of functional significance allowed us to distinguish major biological functions of the DEGs under short- and long-term N limitations. Regardless of the shoots or the roots under both short-term and long-term N limitations, the transport or transporter category was highly enriched (Figure S1C).

**Figure 9:**
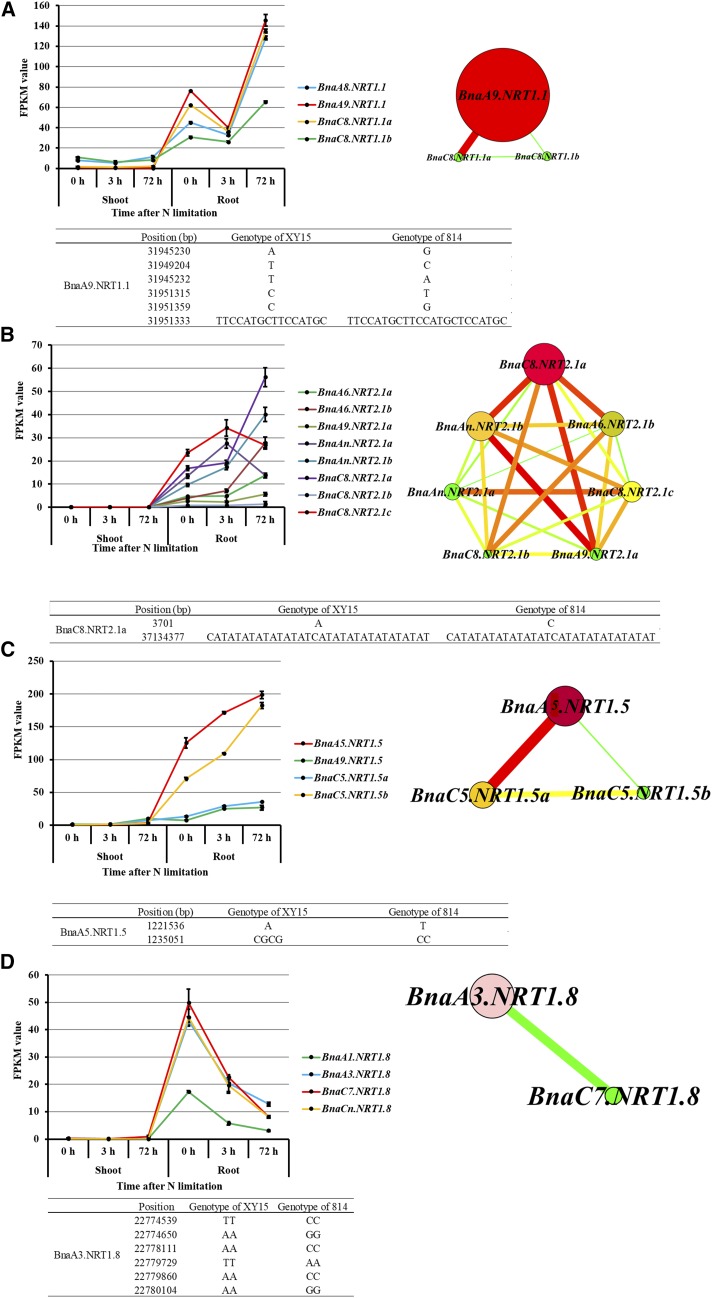
Samples for the transcriptomics-assisted genetic variant identification of some key genes involved in nitrogen use efficiency regulating in *Brassica napus*. Transctiptional expression profiling, gene co-expression network analysis and genetic variants of *BnaNRT1.1s* (A), *BnaNRT2.1s* (B), *BnaNRT1.5s* (C) and *BnaNRT1.8s* (D). For RNA sequencing, the rapeseed seedlings were cultivated under high NO_3_^-^ (6.0 mM) for 10 d, and then transferred to low NO_3_^-^ (0.30 mM). At 0 h, 3 h and 72 h, the shoots and roots of the seedlings were individually sampled with three independent biological replicates. RNA-seq was performed on an Illumina Hiseq X Ten platform (Illumina Inc., San Diego, CA, USA), which generated approximate 6.0-Gb of sequencing data with 150-bp paired-end (PE) reads for each sample. Transcript abundances (FPKM values) were determined from the RNA-seq data with the method described by Hua *et al.* (2017). For the gene co-expression network performed by CYTOSCAPE v. 3.2.1, cycle nodes represent genes, and the size of the nodes represents the power of the interrelation among the nodes by degree value. Edges between two nodes represent interactions between genes.

**Figure 10:**
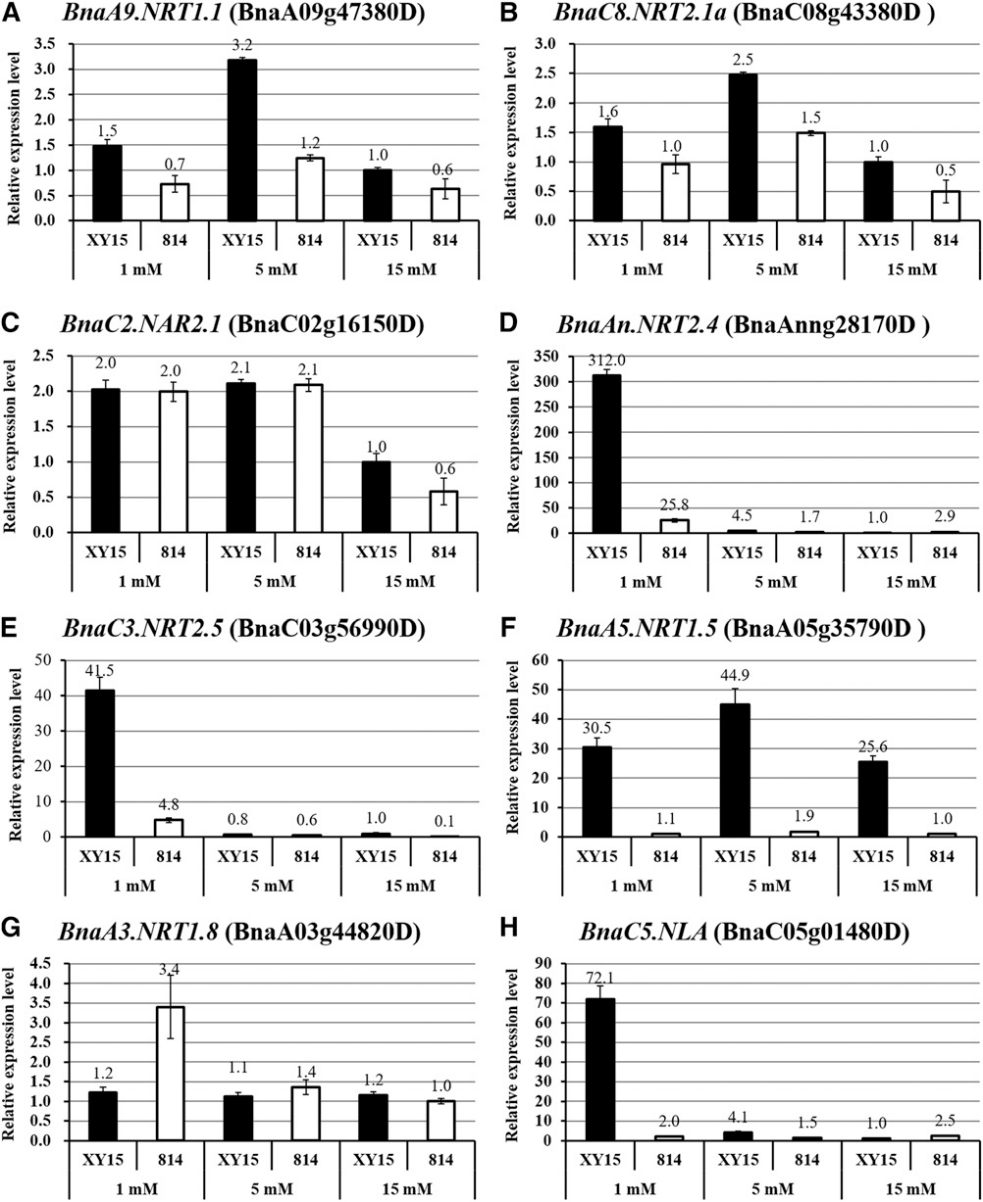
Quantitative reverse-transcription PCR (qRT-PCR) assays of the expression of genes related to N metabolism with genetic variants between the N-efficient genotype “XY15” and the N-inefficient genotype “814”. The relative expression levels of *BnaA9.NRT1.1* (A), *BnaC8.NRT2.1a* (B), *BnaC2.NAR2.1* (C), *BnaAn.NRT2.4* (D), *BnaC3NRT2.5* (E), *BnaA5.NRT1.5* (F), *BnaA3.NRT1.8* (G) and *BnaC5.NLA* (H) in the roots of *B. napus* seedlings, which were grown under low (1 mM), moderated (5 mM) and high (15 mM) nitrate conditions for 15 d. Columns denote means (n = 3), and error bars indicate the standard error.

To further deduce more promising SNP/InDel variants potentially associated with NUEs, we focused on some key *NRT* family genes (Figure 10) through transcriptomics-assisted gene co-expression network analysis (Table 2), and mapped the variants generated by WGS to them. In the genome of *B. napus*, we identified four *NRT1.1* homologs, named after *BnaA8.NRT1.1* (BnaA08g24900D), *BnaA9.NRT1.1* (BnaA09g47380D), *BnaC8.NRT1.1a* (BnaC08g41560D) and *BnaC8*.*NRT1.1b* (BnaC08g15370D), respectively. First, we identified *BnaA9.NRT1.1* as the core member in this gene family through transcriptomics-assisted gene co-expression network analysis (Figure 9A). Further, the WGS data identified five SNPs and an InDel site occurred in *BnaA9.NRT1.1* between the N-efficient genotype “XY15” and the N-inefficient genotype “814”. The genetic variants contained, all of which were located in the promoter of *BnaA9.NRT1.1* (Figure 9A). In the same way, we found nine *NRT2.1* homologs in the rapeseed genome, and *BnaC8.NRT2.1a* (BnaC08g43380D) was thought to be the key gene of the *NRT2.1* family (Figure 9B). The WGS result showed there were an SNP and an InDel in the gene promoter region (Figure 9B).

**Table 2:** Overview of the high-throughput RNA-seq data of the short- and long-term nitrogen limitation experiments

Sample name		Raw reads	Clean reads	Clean bases	Error rate (%)	Q20 (%)	Q30 (%)	GC content (%)	Mapped reads (%)
Shoot	0 h	59635738	57043591	8.55	0.01	97.1	92.6	47.5	89.6
	3 h	58163929	55612390	8.34	0.02	97.2	92.8	47.6	89.8
	72 h	55942618	53982678	8.1	0.02	96.7	91.6	47.1	89.9
Root	0 h	49108675	47000152	7.05	0.02	97.1	92.8	46.1	86.1
	3 h	44434570	42606356	6.39	0.02	96.9	92.2	45.6	87.3
	72 h	50080180	48523315	7.28	0.02	97	92.5	46.3	87.5

Four *BnaNRT1.5s* (*BnaA5.NRT1.5*: BnaA05g35790D; *BnaA9.NRT1.5*: BnaA09g24330D; *BnaC5.NRT1.5a*: BnaC05g24580D; *BnaC5.NRT1.5b*: BnaC05g28620D) were identified in the rapeseed genome, and *BnaA5.NRT1.5* was identified to be the central member (Figure 9C). Further, WGS revealed that an SNP and an InDel occurred in the promoter of *BnaA5.NRT1.5* between “XY15” and “814” (Figure 9C). Among the four *NRT1.8* homologs, *BnaA3.NRT1.8* (BnaA03g44820D) was considered to be the core gene member, and we found six SNPs occurred in its promoter (Figure 9D).

### Transcriptional identification of the differential expression of the NUE-regulating genes With genetic variants

Given that some genomic variants occurred in the key genes related to N-metabolisms, further, we would like to identify whether these variants lead to changes in their mRNA abundances between the N-efficient and -inefficient genotypes. Some genes randomly selected, including N transporters and N limitation adaptation genes, were subjected to qRT-PCR assays (Table 3). The results showed that the expression levels of both *BnaA9.NRT1.1* and *BnaC8.NRT2.1a* were higher in the roots of “XY15” than those of “814” (Figure 10A, B). However, for the NRT2.1 partner NAR2.1, its expression levels did not show obvious differences between the N-efficient and-inefficient genotypes under both low and moderate NO_3_^-^ supply except under high N conditions (Figure 10C). For *BnaAn.NRT2.4* and *BnaC3.NRT2.5* responsible for efficient NO_3_^-^ uptake and phloem loading, their mRNA levels were much higher than those of in the roots of “XY15” than those of “814”, particularly under low NO_3_^-^ (Figure 10D, E). All of these results above-mentioned revealed that “XY15” had a much stronger capability of efficient NO_3_^-^ uptake in the roots.

**Table 3:** Gene-specific primers used for qRT-PCR assays in this study

Gene name	Forward	Reverse	Amplification efficiency
	(5′-3′)	(5′-3′)	
*BnaA9.NRT1.1*	CAAGAAGTTGATTGGTAGCCCG	GTCCTTTATCGCTGCTTTGTCC	99.32%
*BnaC8.NRT2.1a*	AAAGGTACTGAGGAGCACTATTATGG	GTATTCTGAGGCGGCGTAGC	96.37%
*BnaC2.NAR2.1*	TCAAGAAGCTCCTTTTCGCG	TAAATTCAGGCTCCTTTGTAGCC	97.64%
*BnaAn.NRT2.4*	ACGGGAGATGAAGTGAAGTCG	CAGGTCCAGAAGCCACGAA	94.38%
*BnaC3.NRT2.5*	GATGAGCTCTATGTTCTCCGGA	CAGAGAAGGTCTGAAAGAGACCA	98.41%
*BnaA5.NRT1.5*	GATGAAGTCACGCCTTGCG	GCAATGTTCGGTTGGTAACCC	97.53%
*BnaA3.NRT1.8*	GGGTATGGTGGTTATCAGCCC	CGAAAGGAGCGATCCGAGG	96.30%
*BnaC5.NLA*	TGAAAACTGCAGACCCGTCC	TCTTCCCAGTACTCTCGGCG	99.71%
*BnaEF1α*	GCCTGGTATGGTTGTGACCT	GAAGTTAGCAGCACCCTTGG	100.00%
*BnaGDI1*	GAGTCCCTTGCTCGTTTCC	TGGCAGTCTCTCCCTCAGAT	93.10%

For *BnaA5.NRT1.5*, regardless of high or low NO_3_^-^ conditions, its expression levels in the roots of “XY15” were more 20-fold than those of “814” (Figure 10F). However, in terms of *BnaA3.NRT1.8*, we did not identify its significantly differential expression in the roots between “XY15” and “814”; moreover, the N-inefficient genotype showed higher mRNA levels than the N-efficient genotype (Figure 10G). For *BnaC5.NLA*, under low NO_3_^-^, its mRNA abundances were strongly induced in the roots of both “XY15” and “814”; moreover, the expression level in “XY15” was about 35-fold than that of “814” (Figure 10H), which indicated the stronger adaptation ability of “XY15” to N limitation than “814”.

Taken together, it appeared that some genes implicated in efficient N uptake and transport were significantly differentially expressed between the N-efficient genotype XY15 and the N-inefficient genotype 814. To further pin down the main-effect gene(s) responsible for their differential NUEs, we mapped these differentially expressed genes to the regions of the quantitative trait loci (QTL) regulating NUE that were previously reported. In a previous study, Wang *et al.* (2017) dissected the root morphological traits related to NUE under both high N and low N conditions, identified a main-effect NUE-specific QTL cluster on the A9 chromosome. Based on the finding, we retrieved its physical position via mapping the flanking molecular markers of the NUE-A9 QTL to the rapeseed genomic sequences. Through comparison of the NUE-A9 QTL physical interval and the differentially expressed genes having genetic variations, we found only *BnaA9.NRT1.1* was mapped onto the NUE-A9 QTL region, and it may be a core gene regulating differential NUEs between the N-efficient genotype “XY15” and the N-inefficient genotype “814”.

## Discussion

### Differential genomic distribution of the genetic variants

Large and complex allopolyploid genomes, such as *B. napus*, wheat and cotton, propose an enormous challenge for genomic variant discovery because of the presence of multiple homeologous sequences (Bancroft *et al.* 2011; Lai *et al.* 2012; Fang *et al.* 2017; Stein *et al.* 2017). In addition, the repetitive nature of the polyploid genomes has been one of the major barriers to variant identification.

In this study, through high-throughput, -depth and -coverage WGS of an N-efficient genotype “XY15” and an N-inefficient genotype “814”, we identified 1,449,157 SNPs, 335,228 InDels together with thousands of SVs and CNVs. In terms of their genomic distribution, the four types of variants can be categorized into three groups: (i) SNPs and InDels, (ii) SVs, and (iii) CNVs. For SNPs and InDels, the variant number and density of the A_n_ subgenome were higher than those of the C_n_ subgenome (Figures 2, 4) although the assembled C_n_ sub-genome (525.8 Mb) was obviously larger than the A_n_ sub-genome (314.2 Mb) (Chalhoub *et al.* 2014). The results agreed that the A_n_ sub-genome was more variable than the C_n_ sub-genome (Fletcher *et al.* 2016). On the contrary, for SVs, the variants on the C_n_ sub-genome was more than that of the A_n_ sub-genome (Figure 6). In the case of CNV variants, different from SNPs, InDels and SVs, its number did not show obvious sub-genome bias, evenly distributing on the A_n_ and C_n_ sub-genomes (Figure 7). In general, the SNPs, InDels and SVs but not CNVs were largely dependent on the subgenome variability.

### Variation bias of SNPs in allotetraploid rapeseed genotypes

Transition bias refers to the ratio of transition SNPs to transversion SNPs that were more than the expected value of 0.5. In general, transitions provide easy tolerance from selection pressure as they result into synonymous substitutions, which do not alter the conformational structures of protein unlike transversions (Wakeley 1996). Therefore, transitions are usually favored over transversions. The transition bias phenomenon has also been found in soybean (Lee *et al.* 2016) and rice (Subbaiyan *et al.* 2012; Jain *et al.* 2014) and other plant species (Batley *et al.* 2003). A higher Ts/Tv ratio is also indicative of low level of genetic divergence. These ratios are expected to decline with increasing genetic distance between the comparative genotypes as in due course of time; transversions eliminate the record of frequent transitions (Draisma *et al.* 2001). In our previous study, through WGS of boron-efficient and -inefficient genotypes of rapeseed, we identified the Ts/Tv value was about 1.3 (Hua *et al.* 2016b), which was close to the Ts/Tv result (1.35) of Huang *et al.* (2013). Both of them were close to the Ts/Tv result of 1.36 in the present study. It may be assumed that the Ts/Tv value close to 1.3 may be a common rule in allotetraploid rapeseed.

Non-synonymous SNPs that lead to amino acid changes in the protein product are of major interest. Compared with synonymous SNPs, non-synonymous variations are more likely to lead to functional mutations altering phenotype (Huang *et al.* 2013). Thus, the ratio of synonymous SNPs to non-synonymous SNPs can be used as a molecular clock to mirror the evolution rates of species: the smaller the ratios, the faster the species evolve. In this study, the ratio of synonymous SNPs to non-synonymous SNPs were close to 1.50, which was identical to our previous study (Hua *et al.* 2016b), and this ratio may also be universal in rapeseed genotypes. Non-synonymous variants can lead to severe phenotypic consequences that could be either detrimental or beneficial (Ng and Henikoff 2006; Pham *et al.* 2011). Harmful polymorphisms are mostly discarded during evolution through purifying selection whereas beneficial variations could be fixed leading to differential responses of genotypes to biotic and abiotic stresses (Parida *et al.* 2012).

### Genetic variants underlying differential NUEs in allotetraploid rapeseed genotypes

Through high-throughput WGS of low-phosphate-tolerant and -sensitive rice genotypes, Mehra *et al.* (2015) identified more than two million DNA polymorphisms and further mapped them to key phosphate starvation responsive and root architecture genes. In this research, we mapped some genetic variants between the N-efficient and -inefficient genotypes to N-metabolism compounds and some ATPase complexes involved in the regulation of NUE (Figure 8). Specifically, we employed the gene co-expression network analysis to characterize the core NUE-regulating gene(s), and mapped the genetic variants to them (Figure 9). In agreement with the general trend of genome-wide polymorphisms, most of the variants mapped to the NUE-regulating genes were located in the gene promoter regions, and this result indicated that the variants may influence the expression of NUE-regulating genes mainly through modulating their mRNA levels at the transcriptional level. Finally, we confirmed the effect of variants on gene expression via qRT-PCR assays. The qRT-PCR revealed that most of the NUE-regulating genes, such as NO_3_^-^ transporters and N limitation adaptation-related genes, had much higher transcript levels in the roots of the N-efficient genotype than those of the N-inefficient genotype (Figure 10). Previous studies have shown that the N-efficient (higher NUE) genotypes of rapeseed had much stronger N uptake, xylem loading but with weaker vacuolar N storage relative to the N-inefficient (lower NUE) genotypes (Wang *et al.* 2014; Han *et al.* 2016). In the present research, we found that these processes-related genes indeed had some genetic variants through high-throughput WGS. Therefore, the genome-wide high resolution SNP and InDel sites, discovered from the rapeseed genotypes with differential NUEs, can be used for the development of molecular markers for the identification and functional validation of key NUE-regulating genes. Further, genomics-assisted molecular marker-assisted selection can serve as an efficient tool for the identification of elite crops germplasms and genetic improvement of key agronomic traits in crops.
